# PG-Mamba: An Enhanced Graph Framework for Mamba-Based Time Series Clustering

**DOI:** 10.3390/s25165043

**Published:** 2025-08-14

**Authors:** Yao Sun, Dongshi Zuo, Jing Gao

**Affiliations:** 1 Department of Computer Science and Technology, College of Computer and Information Engineering, Inner Mongolia Agricultural University, Hohhot 010011, China; sunyao1024net@emails.imau.edu.cn (Y.S.); zuods@imau.edu.cn (D.Z.); 2 Inner Mongolia Autonomous Region Big Data Center, Hohhot 010091, China

**Keywords:** time series clustering, spatio-temporal graph, deep neural network

## Abstract

Time series clustering finds wide application but is often limited by data quality and the inherent limitations of existing methods. Compared to high-dimensional structured data like images, the low-dimensional features of time series contain less information, and endogenous noise can easily obscure important patterns. When dealing with massive time series data, existing clustering methods often focus on mining associations between sequences. However, ideal clustering results are difficult to achieve by relying solely on pairwise association analysis in the presence of noise and information scarcity. To address these issues, we propose a framework called Patch Graph Mamba (PG-Mamba). For the first time, the spatio-temporal patterns of a single sequence are explored by dividing the time series into multiple patches and constructing a spatio-temporal graph (STG). In this graph, these patches serve as nodes, connected by both spatial and temporal edges. By leveraging Mamba-driven long-range dependency learning and a decoupled spatio-temporal graph attention mechanism, our framework simultaneously captures temporal dynamics and spatial relationships and, thus, enabling the effective extraction of key information from time series. Furthermore, a spatio-temporal adjacency matrix reconstruction loss is introduced to mitigate feature space perturbations induced by the clustering loss. Experimental results demonstrate that PG-Mamba outperforms state-of-the-art methods, offering new insights into time series clustering tasks. Across the 33 datasets of the UCR time series archive, PG-Mamba achieved the highest average rank of 3.606 and secured the most first-place rankings (13).

## 1. Introduction

Research on time series clustering has gained popularity among academics because of its capacity for identifying patterns in the evolution of data and analyzing its intrinsic characteristics. However, the inherent mistakes and trends in time series data, such as periodicity and seasonality, surely present difficulties for clustering tasks. As a result, identifying important features in time series data and creating reliable representations have become important study areas that need more exploration. There are many different clustering methods available today, which we have intuitively divided into the following three primary categories: raw data-based methods, feature-based methods, and deep learning-based methods.

Raw data-based methods. As is well known, K-means [1] is appropriate for large-scale datasets due to its straightforward logic and significant cluster interpretability. However, Euclidean distance cannot be used to quantify the noise and scale variance found in time-series data. To accommodate time series data of different lengths or data of the same length but irregular in time, dynamic time warping (DTW) is applied. For example, Petitjean et al. [2] proposed Dynamic Time Warping Center Average (DBA) based on the concept of global averaging of cluster centers, addressing the issue that DTW aligns two time series data but cannot derive a global average as the cluster center. In addition, Yang et al. [3] proposed the K-Spectral Centroid (KSC). By varying scaling during clustering, KSC presents a novel distance computing method with appropriate translation and scaling factors to capture the time series data’s evolutionary processes. Instead of using DBA to choose average sequences at random, k-shape, a center-of-mass computation method that preserves translation invariance, was developed [4]. K-shape maintains a constant time series while calculating the Shape-Based Distance (SBD) by translating the other time series to determine how similar two time series at different time offsets are. The clustering center is determined to be the time series that is most similar to all the sequences. According to experiments, k-shape is less time-consuming than more sophisticated methods like DTW while remaining competitive.

Feature-based methods. Feature-based methods use conventional clustering methods to complete the clustering task while transforming the original features. Similarity Preserving Representation Learning (SPIRAL), for instance, was proposed by Lei et al. [5] and demonstrates that a similarity matrix may accurately mimic a low-rank matrix. Li et al. [6] proposed a novel clustering method named Symbolic Pattern Forest (SPF), which differs from conventional feature-based approaches. SPF corrects the Symbolic Pattern Tree (SPT) in a global viewpoint by biasing to cluster time series data with linear complexity, while SPT uses randomly chosen patterns to classify time series data in order to capture temporal properties in a local perspective. In conclusion, it is easy to see that scholars concentrate on cutting down on time. However, the features we extract play a major role in the clustering performance of time series data, and we might not obtain satisfactory results if we are unable to assess the time series-specific patterns and capture the deeper feature dependencies. Thus, time series data application to neural networks for deep learning has received increased attention.

Deep learning-based methods. The broad application of neural networks in the analysis of time-series data has led to the gradual emergence of a fundamental problem: how to successfully maintain the time series’ essential characteristics while training the model [7]. According to Guo et al. [7], clustering loss may cause the data’s feature space to be perturbed, producing meaningless feature representations and lowering clustering accuracy. As a result, the Improved Deep Embedded Clustering (IDEC) method was proposed. Experiments showed that the method’s performance is significantly enhanced by the introduction of a denoising encoder, which guarantees that the data with its original distribution during clustering training is only adjusted by clustering loss. For many years, there has been a lot of interest in applying end-to-end modeling to time series clustering. This allows for the comprehensive collection of more effective features in time series data by combining feature extraction with model training. In order to capture spatial and temporal feature relationships, Madiraju et al. [8] proposed Deep Temporal Clustering (DTC), which uses a bi-directional LSTM (BiLSTM) and a one-dimensional Convolutional Neural Network (1D CNN) as encoder layers. Furthermore, Minirocket [9] was proposed by Dempster et al. by replacing the random initialization of convolutional weights with specified rules, which significantly increases the model’s efficiency. Motivated by ROCKET [10], Jorge et al. [11] suggested R-Clustering as a mathematical computation to model the convolution process. This minimizes the time consumption of deep clustering and applies the convolution structure with random weights to time series for the first time. RandomNet is a further implementation of the concept of random weights [12]. RandomNet clusters separately using a combination of multi-branch CNN and LSTM modules and then integrates the multi-branch clustering using Hybrid Bipartite Graph Formulation (HBGF) [13] to generate the final clustering labels.

A growing number of methods based on the Mamba have been proposed in recent years [14], including clustering models [15,16], detection models [17,18] based on image and audio data, and prediction models based on time series data. The potential of the Mamba architecture in distant sensing change detection applications was initially examined by Chen et al. [17]. Additionally, they created a number of decoder designs for a variety of applications, like change detection and semantic recognition, based on the visual Mamba architecture. Zhang et al. [18] quickly learned features from many channels and applied frequency domain features to filter high-frequency features while successfully detecting tiny cracks using a cross-visual Mamba feature extraction module. The potential and effectiveness of Mamba in capturing global context in various task modes are essentially feedback from all of these methods. It is worthwhile to consider how to more effectively apply Mamba’s parallel operation capacity for the time series clustering work, as the clustering task for time series data is not observed. Furthermore, we discovered that the aforementioned CNN-based clustering methods concentrate on the relationship between samples and capture the spatial dependency of time series features, whereas other modules capture the temporal dependency. This separated learning approach frequently fails to learn temporal dependency effectively and ignores spatio-temporal concordance and dynamics. To solve these issues, we first investigate the possibilities of Mamba in time-series clustering tasks. Taking into account the dynamics and non-smoothness of spatio-temporal graph (STG) data [19], we propose PG-Mamba. By transforming time series into patch-based spatio-temporal graph structures and employing Mamba-driven state-space models to learn hierarchical representations of time series for clustering, the framework avoids concentrating on the links between time series.

### Contributions of the Paper

Unlike conventional learning representation methods, we focus on the characteristic patterns of time series rather than the correlation between samples. Specifically, each time series is divided into multiple patches, which are then transformed into a two-dimensional feature matrix. In each graph, a patch is regarded as a graph node, with its corresponding vector representing the feature of that node. To synergistically capture the temporal and spatial characteristics of time series, spatial edges are designed using the K-nearest neighbor algorithm, while adjacent patches serve as temporal edges, thus transforming the time series into an STG. For the first time, we explore spatiotemporal patterns within a single sequence, addressing the issue that traditional methods fail to capture non-adjacent similar patterns.

We designed a module for representation learning. The module leverages Mamba to capture long-range dependencies across patches and applies these global dependencies to a decoupled STG attention module. This approach facilitates the undisturbed learning of local evolution and repetitive patterns in time series data. Finally, we introduce a ternary feature fusion mechanism that integrates the global relationships extracted by Mamba, the local evolution captured by temporal graph attention, and the global repetitive patterns identified by spatial attention. The fused features are then mapped to obtain the final latent representation.

In addition, to bolster the framework’s resilience to noise in time series data, we introduce noise and generate augmented data versions using random masks. A contrastive loss function is then employed to enhance the framework’s denoising capabilities.

To mitigate the interference of clustering loss with the time-series feature space, we propose integrating adjacency matrix reconstruction loss into the clustering process, thereby achieving dual graph reconstruction self-supervision. The performance of PG-Mamba was evaluated on the UCR time-series dataset [20] across 11 clustering algorithms. Experimental results demonstrate that PG-Mamba achieves optimal performance.

## 2. Materials and Methods

**Figure 1:**
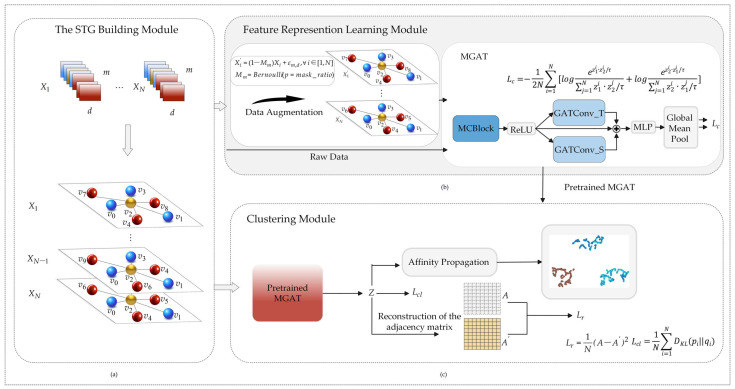
PG-Mamba workflow. (**a**) The STG building module: splitting the time series into equal-sized patches, using the patches as nodes, and then using the proposed temporal edges—where the blue nodes are located—and spatial edges—where the red nodes are located—to build the STG associated with each time series. (**b**) Feature representation learning module. To improve the denoising capabilities of the framework, the augmented time series are fed into the MGAT module together with the source data. (**c**) Clustering module: training the framework to jointly learn spatiotemporal features by calculating the reconstruction loss and clustering loss to obtain a possible representation of the time series.

**Figure 2:**
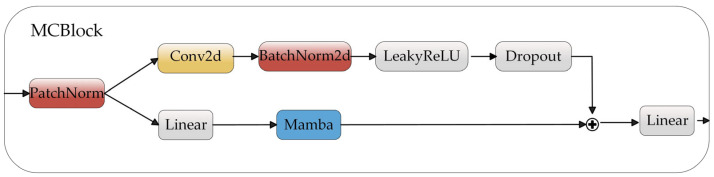
MCBlock module detailed process.

PG-Mamba is a deep clustering framework that builds time series as STG while paying close attention to spatio-temporal dynamics and concordance, thereby changing the conventional approach of viewing time series as a one-dimensional flow and pioneering the concept of a single-sequence internal STG. In the meantime, the multi-level Mamba modules that have been built enable shared pattern learning across patches. Figure 1 shows the overall structure of the framework, which includes three modules: (a) The STG building module, (b) feature representation learning module, and (c) clustering module. Among these, Figure 2 specifically displays the MCBlock module in (b).

### 2.1. Datasets

We evaluated PG-Mamba’s performance using 33 datasets from the popular UCR time series archive, which consist of 13 data types, including device, image, sensor, simulated, spectro, traffic, motion, ECG, EOG, EPG, HAM, hemodynamics, and spectrum. Each dataset contains a minimum of 17 and a maximum of 3601 samples in the train set, and a minimum of 28 and a maximum of 4000 samples in the test set. Furthermore, the time series length varies from 15 to 2844, and the number of time series classes ranges from 2 to 52. Specific information for these datasets is presented in Table 1.

### 2.2. The STG Building Module

We denote the time series as $X\;=\;\{x_{1,}x_{2,\;\ldots ,}x_{n}\}$, where *n* is the number of time series samples. Following Zhang et al. [21] and Abdel-Sater et al. [22], we divide the time series into patches and divide each item into a two-dimensional feature matrix in order to completely capture the time series’ deeper trends. Zhang et al.’s method treats the time series as essentially a one-dimensional sequence, merely preserving local information through segmentation. Abdel-Sater et al. reshape a time step of length *L* into multiple patches to satisfy the model’s input requirement. In contrast, our approach aims to transform the time-series data from a one-dimensional sequence into a graph structure. Using $x_{1}$ as an example, we divide $x_{1}\;={\;\{x_{1i}\}}_{i=1}^{t}$ into $X_{1}^{\prime }$, where *t* is the feature dimension. In this process, a sliding window strategy is adopted. Specifically, unfold is applied with a window size *d* and stride *s* in the feature dimension. As a result, $m\;=\;\left\lfloor (t-d)/s\right\rfloor \;+\;1$ patches are extracted. Therefore, $X_{1}^{\prime }$ is expressed as follows:

(1) $$X_{1}^{\prime }=\left[\begin{array}{ccc} x_{11} & \ldots & x_{1d}\\ \vdots & \ddots & \vdots \\ x_{m1} & \ldots & x_{\mathrm{md}} \end{array}\right],$$

where *d* is the dimension of each patch and *m* is the number of patches.

We regard the patches as nodes and the related vectors as the node characteristics, represented by *V* $=\;\left\{v_{p}|p\;=\;1,\ldots ,\;m\right\},{\;v}_{p}\;\in \;R^{d}$, when building an STG G = (V, E) based on each time series. To concentrate on both temporal and spatial aspects of the time series, we build temporal and spatial edges when building edges between nodes. In particular, we created the temporal edge, represented by $E_{t}\;=\;\{(v_{p},v_{p+∆})|∆\;\in \;\{-w,\;w\}\}$, after defining the parameter *w* and extracting each patch’s neighboring patches during the closed interval [−*w*, *w*]. Additionally, spatial edges were constructed by applying k-nearest neighbors (k = *k*) to connect each patch with its *k* most spatially correlated patches, represented by $E_{\mathrm{sp}}\;=\;\underset{p\;\in \;P}{⋃}\left\{\;p,\;q|\;q\;\in {\;N}_{k}\left(p\right)\right\},\;p\;=\;1,\ldots ,\;m$, where $N_{k}\left(p\right)$ is the set of the node *p*’s *k* nearest neighbor nodes. The temporal and spatial edges are combined and de-duplicated to produce the final set of edges *E*. As a result, every graph has node collection V and a maximum of 24 + (m-6) × (2w) temporal edges and m ×k spatial edges. It is important to note that the connection of boundary nodes requires special consideration when constructing the temporal adjacency graph. Specifically, the first three nodes in the sequence have fewer adjacent nodes than others. For instance, the first node has only three subsequent adjacent nodes; therefore, the number of its temporal edges is 3. Similarly, the second and third nodes have temporal edges of 4 and 5, respectively. The same applies to the last three nodes in the sequence. Consequently, the sum of temporal edges for the first and last three nodes is 24, while 2w temporal edges exist for all the remaining nodes. In this way, the concept of a single-sequence internal STG is first introduced.

### 2.3. Feature Representation Learning Module

In this section, we introduce MGAT, a feature representation learning module that enables both local-to-global generalized pattern learning and shared pattern learning over STG patches. First, a brief introduction to State Space Models (SSMs) is given because we use them in this section. SSMs are a framework for modeling the link between input-output sequences, while the updating process’s coefficient matrix is discretized to allow for deeper data analysis. The following ordinary differential equation [23] represents this:

(2) $$h_{t}\;=\;{\tilde{A}}_{t}h_{t-1}+{\tilde{B}}_{t}x_{t},\;y_{t}\;=\;C_{t}h_{t}+{Dx}_{t},$$

where ${\tilde{A}}_{t}$ is a discretized representation of the state matrix ***A***, ${\tilde{B}}_{t}$ is a discretized representation of the input projection matrix ***B***, and Δ is a time-step discretization factor. The specific representations are as follows:

(3) $${\tilde{A}}_{t}\;={\;e}^{\Delta A},\;{\tilde{B}}_{t}\;=\;{\left(\Delta A\right)}^{-1}(e^{\Delta A}\;-I)\Delta B.$$

This selective scanning mechanism is also seen in Mamba, which focuses on dynamic learning. In particular, Mamba’s Δ, ***B***, and ***C*** are separated following linear mapping of the input data. Δ and ***B*** can dynamically adjust the key information of the input data, and ***C*** can determine which aspects of the state are important to the output. Every training process concentrates more on the initial data patterns and employs real-time hidden states to fully understand the contextual relationships. The model’s comprehension of dependencies is enhanced by this method of selective focus, memorization, and application of contextual information. Simultaneously, sequence blocks’ and feature blocks’ parallel processing method significantly increases computing throughput and is a reliable sequence model.

The Mamba model principle is now fully realized, and we go back to our feature representation learning module. Before entering the MCBlock module, a STG feature first goes through a linear layer that changes the feature dimension *d* to the hidden dimension *D*, converting $X\;\in {\;R}^{n\times m\times d}$ to $X\;\in {\;R}^{n\times m\times D}$. It then enters a multi-layer MCBlock, where the operations are specifically represented as follows for each layer:

(4) X = ReLU(MCBlock(X)).

The graph node features are initially put through the normalization process when they arrive at the MCBlock module. It is important to note that we carry out an independent normalizing operation on the patch features, which maintains the distributional disparities between the patches, in contrast to the prior normalization process. The following is the particular representation:

(5) $$X_{b,d,m}^{\prime }\;=\;\frac{X_{b,d,m}\;-\;\mu_{b,d}}{\sigma_{b,d}},\;\mu_{b,d}\;=\;\frac{1}{M}\sum_{m=1}^{M}X_{b,d,m},\;\sigma_{b,d}\;=\;\sqrt{\frac{1}{M}\sum_{m=1}^{M}{{(X}_{b,d,m}\;-\;\mu_{b,d})}^{2}\;+\;\varepsilon \;},$$

where ε is a hyperparameter that guarantees stable values, we followed the common practice for normalization layers in deep learning and set the value to 1 × 10^−5^. Additionally, we perform a radial transformation to the normalized feature representation, which can be represented as follows, to improve the framework’s expressive power:

(6) $$X_{b,d,m}\;={\;\gamma }_{m}\cdot X_{b,d,m}^{\prime }\;+{\;\beta }_{m},$$

where $\gamma_{m}$ and $\beta_{m}$ are the learnable weights and biases, respectively.

After normalizing the node data, we may divide it into convolution modules and the Mamba module. To begin, we use the convolution module to capture local patterns within patch interiors. Equation (7) provides the specifications for the following convolution module:

(7) X= Dropout(LeakyReLU(BatchNorm2d(Conv2d(X)))).

Concurrently, the STG is fed into the Mamba module to capture long-range dependencies across patches. Subsequently, the node representations from convolution modules and the Mamba module are concatenated. This design is motivated by two key aspects: (1) the Mamba module focuses on learning higher-order node representations, intentionally abstracting away localized fine-grained details at this stage; (2) conversely, convolution modules preserve these detailed features. The concatenation of these representations thus enriches feature diversity by combining complementary information.

Based on the learned global features, spatio-temporal decoupling within a single sequence is performed. Specifically, GATConv is applied to the temporal and spatial edges of the STG, denoted as GATConv_T and GATConv_S, respectively. GATConv_T captures changes in adjacent patches, representing a short-term pattern, while GATConv_S captures similar, non-adjacent patches, representing a long-term pattern. This spatio-temporal decoupling architecture allows for a greater focus on local evolution or repetitive patterns, enabling the capture of key information within the time series.

Meanwhile, we propose a ternary feature fusion mechanism that integrates the features extracted by MCBlock with those obtained after spatio-temporal edge learning. This fusion mechanism enables more effective learning of global feature dependencies, local-to-global dynamics, and pattern repetition consistency. Finally, the ternary features are processed by a multilayer perceptron (MLP), followed by sample-wise global pooling to obtain the latent representation of the time series.

### 2.4. Feature Representation Learning Pre-Training

To mitigate the effects of endogenous noise in time series, data augmentation is performed by injecting adversarial noise. Specifically, we generate a Bernoulli-distributed mask vector $m_{m}\;\sim \;\mathrm{Bernoulli}(p\;=\;\mathrm{mask}\text{\_}\mathrm{ratio})$, where each element independently indicates patch selection with a fixed probability mask_ratio. The input data $X_{1}^{\prime }\;\in \;R^{m\times d}$ is then randomly masked and Gaussian noise is injected, yielding the augmented time series ${\tilde{X}}_{1}^{\prime }$:

(8) $${\tilde{X}}_{1}^{\prime }\;={\;X}_{1}^{\prime (\mathrm{mask})}\;+\;\varepsilon_{m,d},\;X_{1}^{\prime (\mathrm{mask})}\;=\;(1\;-\;m_{m})X_{1}^{\prime },\;\varepsilon_{i,j}\;\sim \;N(0,\sigma^{2}),\;\forall i\;\in \;[1,m],\;j\;\in \;[1,d],$$

where σ denotes the variance and is a predefined hyperparameter. Given that we have standardized the data, resulting in a standard deviation of approximately 1, the morning scale is set to σ=0.1.

The pre-training objective aligns original samples with their augmented counterparts, equipping the framework with discriminative capability against unrelated samples and robustness to noise. Accordingly, we formulate the contrastive loss function (Equation (9)). During pre-training, we further implement early stopping to ensure computational efficiency.

(9) $$L_{c}=-\frac{1}{2N}\sum_{i=1}^{N}[\log\frac{e^{Z_{1}^{i}\cdot Z_{2}^{i}/\tau }}{\sum_{j=1}^{N}Z_{1}^{i}\cdot Z_{2}^{j}/\tau }+\log\frac{e^{Z_{2}^{i}\cdot Z_{1}^{i}/\tau }}{\sum_{j=1}^{N}Z_{2}^{i}\cdot Z_{1}^{j}/\tau }],$$

where N denotes the number of samples, $Z_{1}$ denotes the feature representation learned from the source data, $Z_{2}$ denotes the feature representation learned from the augmented data, and $\tau \;\in {\;R}^{+}$ denotes the temperature hyperparameters. We choose τ = 0.1 to match the standard deviation of the injected noise, thereby maintaining robustness to augmented data.

### 2.5. Cluster Module

The clustering process lies in enhancing the intra-cluster closeness and widening the inter-cluster distance of samples with similar representation patterns. In each training process, the probability distribution q of the potential representation belonging to each cluster center is calculated. And q is normalized to obtain p. KL scattering is applied to quantify the difference between the two probabilities, respectively, pushing ***q*** towards a sharper probability distribution. The clustering loss function is shown in Equation (10).

(10) $$L_{\mathrm{cl}}=\frac{1}{N}\sum_{i=1}^{N}D_{\mathrm{KL}}(p_{i}\left|\right|q_{i}).$$

In addition, inspired by Guo et al. [7], in order to avoid the possible perturbation of the feature space caused by the clustering loss, we propose a new loss function based on the STG, i.e., the loss of neighbor matrix reconstruction, as shown in Equation (11). Based on the potential representation of the framework output, the source and target node features are concatenated, and the reconstruction of the STG adjacency matrix is realized by MLP mapping to a single feature.

(11) $$L_{r}=\frac{1}{N}{\left(A-A_{\mathrm{recon}}\right)}^{2},$$

where N denotes the number of samples and A is the original adjacency matrix.

The loss function of the final framework is represented as follows:

(12) $$L\;={\;L}_{\mathrm{cl}}\;+{\;L}_{r}.$$

### 2.6. Cluster Validity Indices

We used two clustering performance metrics, the Rand Index (RI) and the Adjusted Rand Index (ARI). The RI computes the agreement between predicted cluster assignments and ground-truth cluster assignments by comparing the co-membership of the sample pairs. The ARI [24] extends this by correcting for chance agreement through expectation normalization, using the expectation of a random assignment of the RI to make the statistic expected to have a value of 0.

## 3. Results

### 3.1. Training Setting

PG-Mamba makes full use of the temporal and spatial features of time series and employs contrast loss to align the source data with the augmented data with noise in the pre-training process to train the denoising stability of the representation learning module. We adopt the AdamW optimizer and set both the learning rate and weight decay to 1 × 10^−4^. In addition, an early stopping mechanism is set to enhance training efficiency. In the clustering module, we adopted the Adam optimizer and set the learning rate to 1 × 10^−4^. The clustering is dynamically corrected by the fusion of the KL scatter loss and the reconstruction loss, which maintains the distribution of the original time series during the clustering process and maximizes the advantages of combining the two types of losses. PG-Mamba is trained and optimized on a GPU (Tesla V100S-PCIE32GB) with 3.6 M parameters. For the other method implementations, we follow their respective architectures and hyperparameter values.

### 3.2. Experimental Results

To evaluate the ability of PG-Mamba in time-series clustering, we compared it with 11 more advanced methods. The specific details of each method are listed below:

- K-Means [1] accomplishes the clustering task by assigning samples to the nearest clusters by calculating the Euclidean distance and continuously updating the cluster centers.
- DBA [2] flexibly aligns the time series to the average series based on DTW path backtracking by calculating the DTW distance from each time series to the average series.
- KSC [3] takes into account that the time series should still maintain the same shape when the time axis is panned and captures the evolutionary pattern of the time-series data through different scaling methods when clustering.
- K-shape [4] aligns the nearest clusters by SBD values and uses Rayleigh Quotient maximization to find the center of the representation clusters that have the greatest similarity to all time series.
- SPIRAL [5] obtains the similarity matrix based on the DTW distance of paired time-series data and solves it to obtain new features to accomplish the clustering task using K-Means [1].
- SPF [6] converts the timing data into symbol patterns after time window division and randomly selects the symbol patterns with SPT, which together form the SPF to correct the deviation of SPT in a global view.
- IDEC [7] fine-tunes the clustering loss by pre-training a denoising self-encoder to preserve the distribution of the data.
- DTC [8] uses CNN and BiLSTM as encoder layers to capture spatial and temporal feature dependencies independently.
- Minirocket [10] determines the initialization of the weighted convolution kernel by means of predefined rules to simulate the convolution operation in a linear computation to achieve the classification task.
- R-Clustering [11] simulates inflated convolution to construct a multi-feature representation of each sample by combining inflated values with a convolution kernel. The learned diversity features are dimensionalized by PCA, and clustering is accomplished using K-Means [1].
- Randomnet [12] utilizes a combined module of multi-branch CNN and LSTM to independently extract temporal and spatial features of time-series data and integrates them through multi-branch clustering to obtain the final clustering labels.

We compared the above 11 methods with PG-Mamba based on RI metrics, and the experimental results are shown in Table 2. We bolded the best results and calculated the average RI, the average ranking, and the number of best RIs for each method. If the average ranking has duplicate values, we adopt the averaging rule. Simply put, when there are three values that are all the same and should be ranked from two to four, all three values are standardized to three, and so on. The experimental results show that PG-Mamba achieves the highest average RI (0.736) and average ranking (3.606). It secured first place in RI on 13 datasets—outperforming the second-best method, which attained only three first-place RI rankings and an average ranking of 4.379. Notably, PG-Mamba exhibits more prominent performance on long sequence datasets. It also demonstrates a distinct advantage in high-noise datasets such as AllGestureWiimoteX and EOGHorizontalSignal. Further experiments were conducted on datasets with sequence lengths greater than 500, revealing that the average RI reached 0.781, exceeding the overall 0.736, and the average ranking was 2.71, surpassing the second-best score of 4.03.

**Figure 3:**
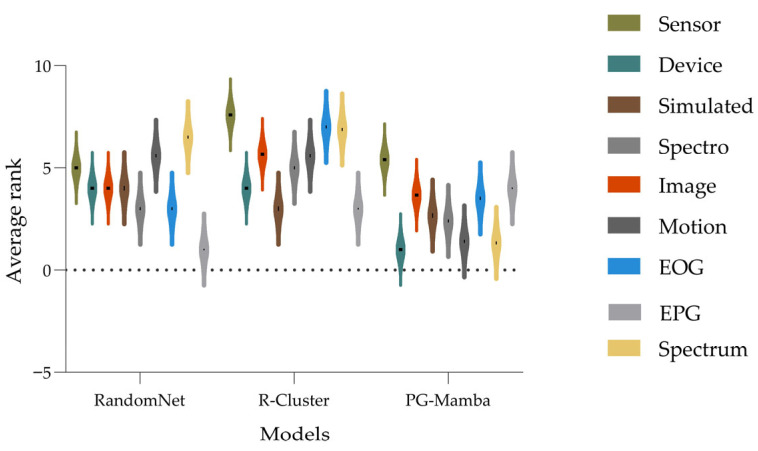
Violin plots on the average RI ranking metric for nine data types.

In addition, to mitigate dataset-specific randomness and category bias, we categorized all data into 10 distinct time series types, and the experimental results are shown in Table 3. It reports the average RI rankings per category (lower values indicate superior clustering), along with each method’s overall average ranking and first-place count. Addi-violin plots for 9 categories (Figure 3) visualize RI ranking distributions, assessing the impact of time series type on method performance. Specifically, because the distribution cannot be effectively represented when a category contains only one dataset, we excluded it from Figure 3. PG-Mamba demonstrates statistically significant superiority across time series dataset types, achieving the highest average RI ranking and the most first-place RI results among all baselines. This robust performance confirms that PG-Mamba’s STG representation learning effectively captures critical features.

**Figure 4:**
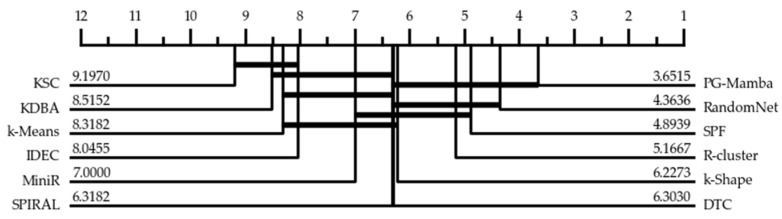
Critical difference diagram of PG-Mamba with advanced methods based on RI.

Meanwhile, in order to facilitate a clear comparison of whether the various algorithms have significant differences on the full dataset, we plotted a key difference plot [25], as shown in Figure 4. The key difference plot calculates the global algorithmic difference by the Friedman test and calculates the *p*-value by combining the Wilcoxon test of two-by-two algorithms in order to assess the statistical significance of each pair of algorithms on the RI metrics. The horizontal line in the figure is used to connect algorithms without significant differences, and the value is the average ranking of the algorithm across all datasets. Results confirm PG-Mamba’s unique dominance (avg. rank:3.6515).

**Figure 5:**
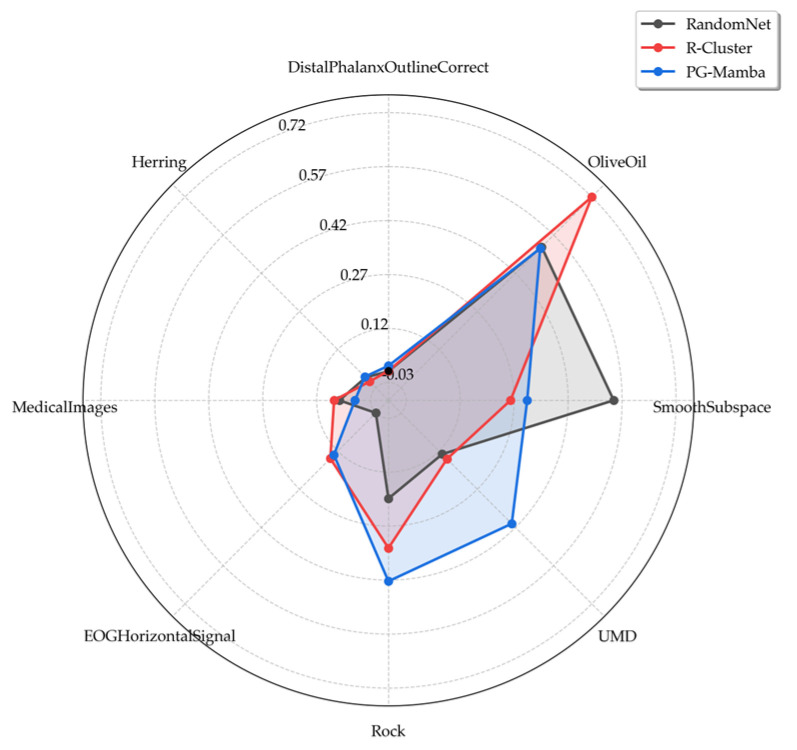
ARI comparison of PG-Mamba with two methods on eight datasets.

**Figure 6:**
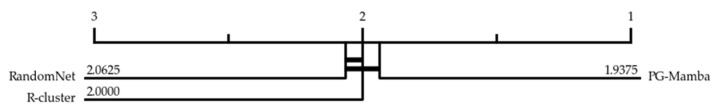
Critical difference diagram of PG-Mamba with two methods based on ARI.

While the RI-based experimental results demonstrate the advantages of PG-Mamba, we also consider the effect of random assignment on chance consistency. We selected two newer clustering methods, R-Clustering [11] and RandomNet [12], eight datasets were randomly selected for plotting circular spider web diagrams based on the ARI, as shown in Figure 5. The figure shows that PG-Mamba never displays an ARI value less than 0. Notably, these datasets are all from different data types, indicating that PG-Mamba’s advantage lies in its ability to capture the data structure rather than random assignment. Additionally, Figure 6 demonstrates that the three algorithms differ significantly and that PG-Mamba performs better than the other two, with an average ARI ranking of 1.9375. In conclusion, PG-Mamba learns the important patterns in the time series and achieves global modeling while maintaining details. This results in a fresh perspective for the time series clustering task.

### 3.3. Analyzing Parameter Sensitivity

**Figure 7:**
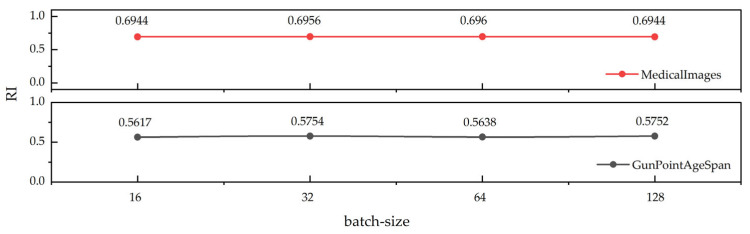
Variation in RI metrics of PG-Mamba with batch-size.

The clustering procedure optimizes cluster separation through the joint minimization of intra-cluster distances and maximization of inter-cluster distances. A robust clustering framework exhibits minimal performance variance under parameter perturbations while maintaining consistent clustering quality. To validate the robustness of the proposed framework, we conducted parameter sensitivity analyses across three heterogeneous time series datasets. Given the implementation of early-stopping mechanisms during training, epoch sensitivity was excluded from consideration. Figure 7 presents the RI metric fluctuations of PG-Mamba under varying batch sizes on the MedicalImages dataset and GunPointAgeSpan dataset, demonstrating its operational stability.

**Figure 8:**
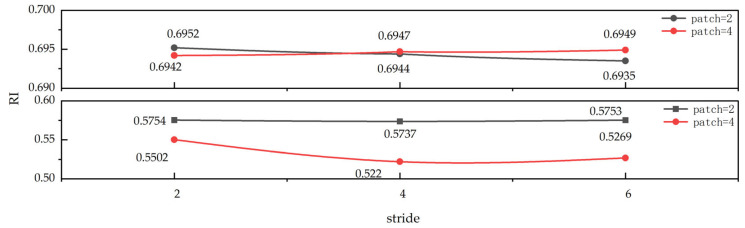
Variation in RI metrics of PG-Mamba with different patches and strides on MedicalImages dataset (top) and GunPointAgeSpan dataset (bottom).

Also, the more important hyperparameters in our framework include patch and stride, we perform a sensitivity analysis on the remaining two datasets as shown in Figure 8. As illustrated in the figure, the MedicalImages dataset (top) and GunPointAgeSpan dataset (bottom), differing by several times in the number of features, were analyzed. With stride values ranging from two to six and patch values from two to four, almost no significant changes in the results were observed. Hyperparameter stability is quantitatively measured using the relative standard deviation (RSD = *σ*/*μ*). After traversing all combinations of patch ∈ {2,4,6} and stride ∈ {2,4,6,8,10,12,18,20}, the RSD for the MedicalImages dataset is only 0.03%, while it is 2.6% for the GunPointAgeSpan dataset. We also observed that increasing the stride from 2 to 20 results in a decrease of only 1.4% in the RI of MedicalImages, whereas the RI of GunPointAgeSpan decreases by as much as 10%. Notably, the RI of GunPointAgeSpan does not exhibit significant fluctuations when the stride is less than or equal to eight. Considering the characteristics of the dataset, the GunPointAgeSpan dataset contains a large amount of sensor noise and domain shift redundancy, and an excessively large stride will skip key information, leading to adverse effects.

**Figure 9:**
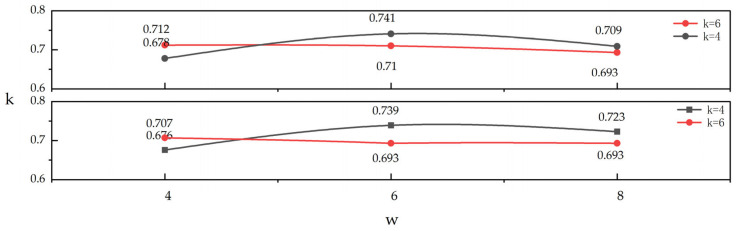
Variation in RI metrics of PG-Mamba with different *k* and *w* on SmoothSubspace dataset (top) and UMD dataset (bottom).

Evidently, the inherent temporal redundancy of different datasets directly determines their sensitivity to the same hyperparameters. In addition, a sensitivity analysis was conducted on the hyperparameters *k* and *w* used for STG construction, as detailed in Figure 9. Specifically, *w* was varied from 4 to 6, and *k* values of 4 and 6 were tested. After ten experimental validations, the results for the SmoothSubspace dataset (top) and UMD dataset (bottom) were obtained. Ultimately, *k* = 4 and *w* = 6 were selected as the parameter values for our framework. In summary, parameter sensitivity analysis allows us to derive the mapping rules between data and parameters, thus offering a valuable reference for minimizing parameter tuning costs across various datasets.

### 3.4. Analyzing the Time Complexity

**Figure 10:**
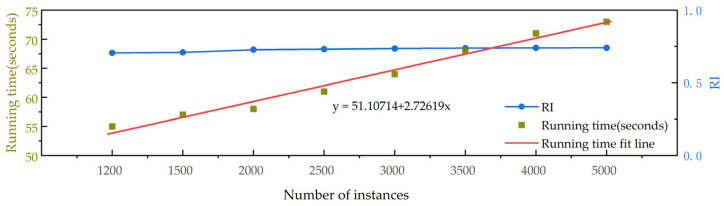
Running time of PG-Mamba on the different number of instances.

In real-world applications, the impact of the dataset sample size on clustering performance is non-negligible. To verify the scalability of the framework, a detailed analysis was conducted on the TwoPatterns dataset, as shown in Figure 10. The number of time series was selected, ranging from 1200 to 5000, to analyze the time complexity and its impact on the RI. As the figure illustrates, our framework maintains a relatively stable RI value without significant fluctuations. Furthermore, the time consumption, when fitted against the timeline, demonstrates a linear time complexity.

### 3.5. Ablation Study

To verify the effectiveness of each component in PG-Mamba, we compared PG-Mamba with three variants on 33 datasets, evaluating them based on the RI metric and the number of floating-point operations (FLOPs), as detailed in Table 4. PG-Mamba w/o Conv signifies the variant with convolution removed, PG-Mamba w/o Mamba signifies the variant with Mamba removed, and PG-Mamba without Conv and Mamba signifies the variant with both removed. As demonstrated in Table 4, the full PG-Mamba outperforms all three variants in terms of the Average Rand Index and exhibits the highest return per unit of computation.

## 4. Discussion

Reviewing the existing clustering methods, more attention is paid to finding the relationship between two time series to accomplish the clustering task. However, the inherent intrinsic noise as well as the low-dimensional nature of time series tend to have redundant data and limited information. Therefore, when faced with massive amounts of time series data, only focusing on the relationship between the two may not be able to obtain better clustering results. This study provides another way of thinking, where we focus on the time series itself, aiming to find the key patterns within it, and clustering the time series data based on the key patterns carried by the time series to achieve a better performance.

To comprehensively capture essential temporal patterns and leverage the full feature potential of time series data, we propose PG-Mamba—a novel framework that advances beyond conventional independent feature learning paradigms. Our method pioneers a collaborative spatio-temporal feature fusion scheme, introducing the concept of single-sequence spatio-temporal patterns. Instead of simply treating a time series as a one-dimensional flow, we decouple it into temporal evolution and pattern space through carefully designed temporal and spatial edges. This allows us to construct a spatio-temporal graph structure, enabling dynamic modeling of spatio-temporal consistency. At the core of our architecture lies Mamba-driven long-range dependency learning and decoupled spatio-temporal graph attention. Mamba captures global patterns within the spatio-temporal graph (STG), while spatio-temporal graph attention focuses on key pattern features. Furthermore, a ternary feature fusion mechanism effectively preserves critical information. This research marks the first exploration into the effectiveness of a hybrid Mamba and spatio-temporal GAT architecture for time series clustering tasks.

This framework employs adversarial noise injection to complete the pre-training process, thereby enhancing the framework’s denoising capability. To address potential distribution perturbations caused by the clustering objective, a dual-loss optimization strategy is adopted: (1) KL divergence is utilized for clustering alignment; (2) a novel neighbor matrix reconstruction loss is introduced to maintain the integrity of the spatio-temporal structure. This comprehensive approach ensures both clustering performance and framework stability, while preserving the integrity of the data distribution. We verified that PG-Mamba is not randomized for a particular data type by comparing the average rankings of different time series types. In order to exclude the inflated RI caused by randomness, we also compared ARI with better algorithms on all datasets, and the experimental results showed that PG-Mamba achieved the highest average ranking and average value. Overall, PG-Mamba focuses on the time series itself, learns the key patterns, and fills the gap where the Mamba model is not applied to clustering tasks. This exploration provides a new way of thinking about the time series clustering task.

However, our framework still has some limitations: (1) We are working with manually set hyperparameters, and although it has been shown that PG-Mamba has only small fluctuations for hyperparameter variations, in the future we will find a way to automatically extract hyperparameters that are suitable for a specific type of time series and adaptively accomplish the clustering task. (2) Inspired by the “in-cluster sharing” fine-tuning strategy for edge devices proposed by Abdel-Sater et al. [22], we recognize that techniques such as low-rank fine-tuning can be adopted to reduce the framework’s tuning cost. Therefore, our future work will focus on designing fine-tuning strategies tailored for graph neural networks or time series frameworks to balance clustering effectiveness and framework performance.

## 5. Conclusions

This study introduces PG-Mamba, a deep learning framework specifically designed for time series clustering. In contrast to traditional time feature learning methods, the framework transforms time series into patch-based STG structures and employs Mamba-driven state space models to capture global dependencies. Concurrently, a spatio-temporal GAT module is utilized to decouple spatio-temporal features, ultimately achieving clustering through multivariate feature fusion. To validate PG-Mamba’s performance, 33 datasets were selected from the UCR time series archive and compared against 11 state-of-the-art methods, with RI and ARI used as evaluation metrics. The experimental results demonstrate PG-Mamba’s superior performance in time series clustering tasks. While this study has achieved promising results, we acknowledge that the manual setting of hyperparameters can influence the framework’s performance. In the future, we will focus on designing a method that can automatically learn hyperparameters based on time series characteristics to achieve better clustering performance.

## Figures and Tables

**Table 1:** Descriptions of datasets for experiments.

Dataset Name	Data Type	Train Size	Test Size	Length	Class
AllGestureWiimoteX	Sensor	300	700	500	10
Chinatown	Traffic	20	343	24	2
Coffee	Spectro	28	28	286	2
CricketY	Motion	390	390	300	12
DistalPhalanxOutlineCorrect	Image	600	276	80	2
DodgerLoopGame	Sensor	20	138	288	2
ECGFiveDays	ECG	23	861	136	2
EOGHorizontalSignal	EOG	362	362	1250	12
EOGVerticalSignal	EOG	362	362	1250	12
EthanolLevel	Spectro	504	500	1751	4
FordA	Sensor	3601	1320	500	2
Fungi	HRM	18	186	201	18
GesturePebbleZ1	Sensor	132	172	455	6
GunPoint	Motion	50	150	150	2
GunPointAgeSpan	Motion	135	316	150	2
Herring	Image	64	64	512	2
InlineSkate	Motion	100	550	1882	7
InsectEPGSmallTrain	EPG	17	249	601	3
Meat	Spectro	60	60	448	3
MedicalImages	Image	381	760	99	10
OliveOil	Spectro	30	30	570	4
Phoneme	Sensor	214	1896	1024	39
PigAirwayPressure	Hemodynamics	104	208	2000	52
RefrigerationDevices	Device	375	375	720	3
Rock	Spectrum	20	50	2844	4
ScreenType	Device	375	375	720	3
SemgHandMovementCh2	Spectrum	450	450	1500	6
SemgHandSubjectCh2	Spectrum	450	450	1500	5
SmoothSubspace	Simulated	150	150	15	3
UMD	Simulated	36	144	150	3
Wine	Spectro	57	54	234	2
Worms	Motion	181	77	900	5
TwoPatterns	Simulated	1000	4000	128	4

**Table 2:** Comparison of PG-Mamba with state-of-the-art methods.

Datasets	Data Type	k-Means	KSC	k-Shape	SPF	SPIRAL	KDBA	IDEC	DTC	MiniR	RandomNet	R-Cluster	PG-Mamba
AllGestureWiimoteX	Sensor	0.83	0.099	0.854	0.836	0.835	0.819	0.824	0.817	0.82	0.834	0.812	**0.878**
Chinatown	Traffic	0.527	0.526	0.526	0.633	**0.787**	0.569	0.513	0.527	0.582	0.592	0.579	0.489
Coffee	Spectro	0.751	0.805	0.751	0.834	0.805	0.777	0.492	0.492	0.75	**1**	0.777	0.815
CricketY	Motion	0.854	0.514	0.876	0.871	0.856	0.805	0.858	0.848	0.869	0.868	0.876	**0.884**
DistalPhalanxOutlineCorrect	Image	0.499	0.499	0.499	0.5	0.501	0.502	**0.526**	0.521	0.501	0.501	0.500	0.499
DodgerLoopGame	Sensor	0.503	**0.639**	0.585	0.517	0.529	0.515	0.556	0.597	0.502	0.529	0.510	0.513
ECGFiveDays	ECG	0.5	0.871	**0.879**	0.578	0.53	0.511	0.506	0.596	0.761	0.531	0.512	0.507
EOGHorizontalSignal	EOG	0.857	0.422	**0.877**	0.869	0.866	0.797	0.082	0.855	0.569	0.874	0.857	0.859
EOGVerticalSignal	EOG	0.856	0.603	**0.876**	0.87	0.851	0.8	0.855	0.838	0.786	0.861	0.840	0.871
EthanolLevel	Spectro	0.623	0.621	0.623	0.626	0.606	0.553	0.613	0.689	0.621	0.627	0.673	**0.741**
FordA	Sensor	0.5	0.505	**0.578**	0.501	0.5	0.5	0.5	0.51	0.5	0.501	0.503	0.507
Fungi	HRM	0.938	0.794	0.798	0.99	0.993	0.398	0.959	0.926	0.999	0.976	**1.000**	0.930
GesturePebbleZ1	Sensor	0.802	0.213	0.87	**0.904**	0.795	0.75	0.838	0.841	0.832	0.818	0.796	0.826
GunPoint	Motion	0.497	0.507	0.497	0.497	0.498	0.497	0.498	**0.53**	0.497	0.507	0.497	0.512
GunPointAgeSpan	Motion	**0.628**	0.518	0.53	0.514	0.546	0.518	0.499	0.559	0.499	0.499	0.519	0.575
Herring	Image	0.5	0.499	0.504	0.504	0.506	0.499	0.504	0.489	0.502	0.508	0.507	**0.777**
InlineSkate	Motion	0.736	0.76	0.749	0.759	0.693	0.669	0.749	0.763	0.738	0.759	0.749	**0.832**
InsectEPGSmallTrain	EPG	0.564	0.574	0.707	0.732	0.722	0.629	0.628	0.635	0.775	**1**	0.768	0.749
Meat	Spectro	0.785	0.785	0.729	0.83	0.852	0.8	0.328	0.578	0.768	0.86	0.854	**0.872**
MedicalImages	Image	0.665	0	0.668	0.667	0.668	0.684	0.682	0.651	0.678	0.677	0.674	**0.694**
OliveOil	Spectro	0.739	0.845	0.745	0.875	0.872	0.828	0.288	0.288	0.775	0.815	**0.882**	0.816
Phoneme	Sensor	0.911	0.491	0.929	0.93	0.928	0.789	0.922	0.082	0.928	**0.932**	0.929	0.921
PigAirwayPressure	Hemodynamics	0.914	0.016	0.903	0.936	0.96	0.84	0.938	0.883	0.967	**0.969**	0.967	0.925
RefrigerationDevices	Device	0.555	0.332	0.556	0.558	0.587	0.588	0.554	0.518	0.538	0.58	0.578	**0.662**
Rock	Spectrum	0.664	0.38	0.689	0.719	0.657	0.675	0.66	0.722	0.734	0.695	0.747	**0.777**
ScreenType	Device	0.562	0.332	0.557	0.568	0.566	0.525	0.562	0.635	0.559	0.569	0.591	**0.642**
SemgHandMovementCh2	Spectrum	0.735	0.638	0.739	0.756	0.604	0.732	0.743	0.783	0.762	0.743	0.743	**0.822**
SemgHandSubjectCh2	Spectrum	0.734	0.645	0.721	0.734	0.568	0.661	0.73	**0.797**	0.698	0.7	0.696	0.782
SmoothSubspace	Simulated	0.709	0.333	0.682	0.645	**0.896**	0.629	0.585	0.638	0.631	0.816	0.662	0.741
UMD	Simulated	0.557	0.559	0.612	0.622	0.626	0.557	0.614	0.682	0.616	0.621	**0.762**	0.739
Wine	Spectro	0.496	0.496	0.496	0.5	0.495	0.496	0.496	0.496	0.503	0.502	0.507	**0.660**
Worms	Motion	0.646	0.52	0.656	0.683	0.666	0.644	0.263	0.701	0.648	0.687	0.655	**0.724**
TwoPatterns	Simulated	0.628	0.537	0.675	0.693	0.656	**0.945**	0.63	0.705	0.638	0.725	0.743	0.741
Average Rand Index		0.675	0.511	0.695	0.705	0.698	0.652	0.606	0.642	0.683	0.717	0.705	**0.** **736**
Average rank		8.364	9.212	6.303	4.894	6.318	8.545	8.076	6.303	7.030	4.379	4.970	**3.606**
Number Best		1	1	4	1	2	1	1	2	0	4	3	**13**

Best results are highlighted in bold.

**Table 3:** RI of PG-Mamba vs. state-of-the-art methods on 10 distinct dataset types.

Datasets	k-Means	KSC	k-Shape	SPF	SPIRAL	KDBA	IDEC	DTC	MiniR	RandomNet	R-Cluster	PG-Mamba
Sensor	8.80	8.00	**2.40**	3.90	7.00	9.60	6.40	5.80	8.10	5.00	7.60	5.40
Device	7.75	12.00	8.50	8.50	4.50	6.50	8.25	6.50	9.50	4.00	4.00	**1.00**
Image	10.00	11.17	8.17	7.33	5.50	5.17	**3.33**	8.33	5.67	4.00	5.67	3.67
Simulated	8.83	11.33	7.00	6.00	4.33	7.50	9.67	5.33	8.33	4.00	3.00	**2.67**
Spectro	8.20	6.60	8.50	3.80	6.90	7.70	10.70	8.90	7.70	3.60	3.00	**2.40**
Motion	7.80	7.40	6.30	6.30	6.70	10.10	8.60	3.60	8.60	5.60	5.60	**1.40**
EOG	6.00	11.50	**1.00**	3.00	5.50	9.50	9.00	8.50	10.50	3.00	7.00	3.50
EPG	12.00	11.00	7.00	5.00	6.00	9.00	10.00	8.00	2.00	**1.00**	3.00	4.00
HRM	7.00	11.00	10.00	4.00	3.00	12.00	6.00	9.00	2.00	5.00	**1.00**	8.00
Spectrum	7.17	11.33	7.00	4.17	11.67	9.33	7.17	2.33	4.67	6.50	5.33	**1.33**
Average rank	9.05	10.30	6.60	4.95	5.90	8.90	7.95	6.45	7.25	3.25	4.05	**2.70**
Num. Top-1	0	0	2	0	0	0	1	0	0	1	1	**5**

Best results are highlighted in bold.

**Table 4:** Ablation results of PG-Mamba on 33 UCR datasets.

Model Configuration	Average Rand Index	Average FLOPs (G)
PG-Mamba	0.736 (+0.040)	49.851 (+6.828)
PG-Mamba w/o Conv	0.715 (+0.019)	44.751 (+1.728)
PG-Mamba w/o Mamba	0.708 (+0.012)	49.188 (+6.165)
PG-Mamba w/o Conv and Mamba	0.696	43.023

## Data Availability

The original data presented in the study are openly available in https://github.com/aniani6aa/PG-Mamba.
